# Supplementary material for: Anticancer Activity of Snake Venom Against Breast Cancer: A Scoping Review
Source: Toxins (Basel). 2025 Sep 25;17(10):477. doi: 10.3390/toxins17100477 (PMC12567882; doi:10.3390/toxins17100477)
Supplement: Supplementary file 1 [file toxins-17-00477-s001.zip › Supplementary Material S1. Table S1-S4.pdf]

toxins

MDPI

Review

Anticancer Activity of Snake Venom Against Breast Cancer: A Scoping Review

Eun-Jin Kim [1], Jang-Kyung Park [2], Soo-Hyun Sung [3,*] and Hyun-Kyung Sung [4,*]

1 Department of Pediatrics of Korean Medicine, Korean Medicine Hospital, Dongguk University Bundang Medical Center, Seongnam 13601, Republic of Korea; utopialimpid@naver.com
2 Department of Korean Medicine Obstetrics and Gynecology, School of Korean Medicine, Pusan National University, Yangsan 50612, Republic of Korea; vivat314@pusan.ac.kr
3 Department of Policy Development, National Institute of Korean Medicine Development, Seoul 04554, Republic of Korea
4 Department of Education, College of Korean Medicine, Dongguk University, Gyeongju 38066, Republic of Korea
* Correspondence: koyote10010@nikom.or.kr (S.-H.S.); shksolar@dongguk.edu (H.-K.S.)

Table S1. Anticancer Activity of crude snake venom in breast cancer cells.

Author (Year)	Snake Venom	Target Cell, Animal Model	Concentration	Duration	Method	Results
Al-Asmari et al. (2016) [18]	*Bitis arietans*<br>*Cerastes gasperettii*<br>*Echis coloratus*<br>*Echis pyramidum*	MDA-MB-231	5, 10 μg/mL	2 wk	Clonogenic survival assay	-reduction in colony formation [a]
Al-Sadoon et al. (2012) [19]	*Walterinnesia aegyptia*	MDA-MB-231<br>MCF-7	1, 5, 10, 20, 50, 100,1000 ng/mL	1, 2, 6, 12, 24, 48 h	MTT assay<br>Trypan Blue exclusion assay	-decreased cell viability percentage in a dose-dependent manner
Badr et al. (2013) [38]	*Walterinnesia aegyptia*	[Xenograft] BALB/c mice	2.5 μg/kg/d	28 d	Monitored tumor volumes every 3d	-inhibited tumor growth from days 7 to 28 and 17 to 28 [a]

toxins

	Walterinnesia aegyptia with silica NPs	(10 wk, 22-25g) MDA-MB-231	1 μg/kg/d			-the snake venom with silica NPs was greater than venom alone
Badr et al. (2014) [41]	*Walterinnesia aegyptia*	Human Breast cancer tissue samples	1, 5, 10, 20, 50, 100, 1000 ng/mL	1, 2, 6, 12, 24, 48 h	MTT assay	-decreased cell viability percentage in a dose- and time dependent manner
Erlista et al. (2023) [24]	*Naja kaouthia* (25% methanol peptide fraction)	MCF-7	1-3 μg/mL	24 h	MTT assay	-cytotoxic effect of some of the investigated methanol fractions on the MCF-7 cancer cell line in a concentration-dependent manner<br>-5% methanol fraction had the lowest percentage of viability and showed significant antitumor activity against MCF-7 cells
Jimenez-Canale et al. (2022) [29]	*Crotalus molossus molossus* with chitosan NPs	T-47D	0.98, 1.95, 3.91, 7.81, 15.63, 31.25 mg/mL	24 h	MTT assay	- decreased cell viability in a dose-dependent manner, achieving approximately 30% viability at 31.25 μg/mL<br>-the snake venom with chitosan NPs lower cell viability than whole venom alone
Kisaki et al. (2021) [30]	*Bothrops Jararaca*	MDA-MB-231 MCF-7	0-20 μg/mL	24 h	WST-1 reagent assay	-cell death began at 2.5 μg/mL in MDA-MB-231 and MCF-7 cells<br>-most MCF-7 cells died, while only half of MDA-MB-231 cells were affected at 5.0 μg/mL
					Cell Morphology assay	-morphological changes such as cell shrinkage and cell birefringence change (at >2.5 μg/mL)
Malekara et al. (2020) [32]	*Vipera raddei kurdistanica*	MDA-MB-231 MCF-7	0, 0.62, 1.25, 2.5, 5, 10 μg/mL	24, 48, 72, 96 h	MTT assay	-increased cytotoxicity in a dose and time-dependent manner
			0, 0.31, 0.62, 1.25, 2.5, 5, 10 μg/mL		Trypan Blue exclusion assay<br>LDH assay	

[a] $p < 0.05$; h−hours; d−days; wk−weeks; LDH−Lactate Dehydrogenase; MTT−3-(4,5-dimethylthiazol-2-yl)-2,5-diphenyltetrazolium bromide; NP−nanoparticles; WST-1−Water-soluble tetrazolium salt-1

Table S2. Anticancer Activity of snake venom bioactive proteins in breast cancer cells.

Author (Year)	Snake Venom	Main component	Target cell	Concentration	Duration	Method	Results
Bezerra et al. (2019) [20]	*Bothrops jararacussu*	BthTX-I	MDA-MB-231<br>MCF-7<br>SK-BR-3	0, 12, 25, 51, 102, 204, 409 μg/mL	24 h	MTT assay	-decreased cell viability [a]<br>-SKBR3 cells were more sensitive to BthTX-I than MCF-7 and MDA-MB-231 cells
Bhattacharya et al. (2023) [21]	*Russell's viper*	SPAD-1	MCF-7	0, 2, 20 nM	24, 48 h	Resazurin assay	-reduced cell-to-cell attachment in a dose-dependent manner
						Trypan Blue exclusion assay	-reduced live cell number
						Cell Morphology assay	-decreased cell viability percentage in a dose-dependent manner
Bhowmik et al.(2017) [22]	*Naja kaouthia*	NKCT1 combined with gold NP	MDA-MB-231<br>MCF-7	3.9 μg/mL	24, 48 h	MTT assay<br>(with or without 17- beta estradiol)	-increased percentage of cell death in MCF-7 cells [a] (both absence and presence of 17- beta estradiol) but MDA-MB-231 cells show lower percentage of cell death
Derakhshani et al. (2020) [23]	*Naja naja oxiana*	Recombinant Cytotoxin II	MCF-7	4 μg/mL	24 h	MTT assay	-decreased cell viability percentage in a dose-dependent manner
Gallego-Londoño et al. (2025) [25]	*Crotalus durissus*<br>*Naja atra*	Crotalicidin<br>NA	MDA-MB-231<br>MCF-7	0, 10, 20, 30, 40, 50 μM	24 h	MTT assay	-decreased cell viability percentage in a dose-dependent manner [b]
				Crotalicidin: 12.5, 25, 50 μM<br>NA: 3.125, 6.25, 12.5 μM /mL	6 h	Cell Morphology assay	-decreased size and increased granularity
						LDH assay	-increased LDH release in a dose-dependent manner
Gimenes et al. (2017) [26]	*Crotalus durissus collilineatus*	γCdcPLI	MDA-MB-231<br>MCF-7	1.56, 3.125, 6.25, 12.5, 25, 50 μg/mL	24 h	MTT assay	-inhibited growth of cancer cells in a concentration-dependent manner (MDA-MB-231 cells were more affected than MCF-7 cells)

						LDH assay	-increased LDH activity in a dose-dependent manner
Hiu et al. (2021) [27]	*Naja sumatrana*	CTX-I	MCF-7	2, 4, 6, 8, 16, 32, 64 µg/mL	4, 8, 16, 24 h	LDH assay	-increased LDH activity and membrane permeabilization in a time-dependent manner at lower CTX-I concentrations
Jebali et al. (2014) [28]	*Macrovipera lebetina*	Lebecin	MDA-MB-231	1, 5, 10 µg/mL	48 h	MTT assay	-unable to affect the viability of MDA-MB-231 cells
Latinovi et al. (2017) [31]	*Vipera ammodytes ammodytes*	Dimeric disintegrin	MDA-MB-231	0.005, 0.05, 0.5, 5, 50, 500 nM	0, 24, 48, 72 h	PrestoBlue™ cell viability assay	-decreased cell viability percentage (at ≥ 50 nM)
Silva et al. (2018) [33]	*Bothrops pauloensis*	BnSP-6	MDA-MB-231 MCF-7	6.25, 12.5, 25, 50, 100 µg/mL	24 h	MTT assay	-increased cytotoxicity in a concentration-dependent manner
Van Petten de Vasconcelos Azevedo et al. (2016) [35]	*Bothrops pauloensis*	BnSP-6	MDA-MB-231	6.25, 12.5, 25, 50, 100 µg/mL	24 h	MTT assay	-decreased cell viability percentage in a dose-dependent manner [a]
Van Petten de Vasconcelos Azevedo et al. (2019) [36]	*Bothrops jararacussu*	BthTX-II	MDA-MB-231	1.56, 3.125, 6.25, 12.5, 25, 50, 100 µg/mL	24 h	MTT assay	-increased cytotoxicity in a dose-dependent manner
				50 µg/mL	24 h	LDH assay	-increased LDH activity [b]
Van Petten de Vasconcelos Azevedo et al. (2022) [37]	*Bothrops jararacussu*	BthTX-II	HUVEC	0.781, 1.56, 3.125, 6.25, 12.5, 25, 50 µg/mL	24 h	MTT assay	-decreased cell viability percentage both absence [b] and presence [c] of VEGF stimulation (at ≥ 12.5 µg/mL)
Peña-Carrillo et al. (2021) [43]	*Bothrops marajoensis*	pBmje	MCF-7	50, 250 µM	48 h	MTT assay	- -decreased cell viability percentage [a] (at 250 µM)

[a]–$p<0.05$; [b]–$p<0.001$; [c]–$p<0.0001$; h–hours; d–days; wk–weeks; EGF-epidermal growth factor; LDH–Lactate Dehydrogenase; MTT–3-(4,5-dimethylthiazol-2-yl)-2,5-diphenyltetrazolium bromide; NA-NA-CATH-ATRA-1-ATRA-1; VEGF–Vascular endothelial growth factor

toxins

MDPI

Table S3. Anticancer Mechanism of crude snake venom in breast cancer cells.

Author (Year)	Snake Venom	Target Cell, Animal Model	Concentration	Duration	Method	Results
Al-Asmari et al. (2016) [18]	*Bitis arietans*<br>*Cerastes gasperettii*<br>*Echis coloratus*<br>*Echis pyramidum*	MDA-MB-231	5, 10 μg/mL	24 h	Annexin V binding assay	-increased percentage of early and late apoptotic cells [a]
					Scratch wound healing assay<br>Matrigel invasion assay	-decreased cell motility [a]<br>-decreased Matrigel invasion [a]
					Oxidative stress marker<br>Inflammatory cytokine	-increased ROS production [a]<br>-decreased IL-8 and IL-6 expressions
					Protein expression	-downregulated: RhoC [a], p-Erk1/2 [a]
Al-Sadoon et al. (2012) [19]	*Walterinnesia aegyptia*	MDA-MB-231<br>MCF-7	NR	12h	Annexin V binding assay	-increased percentage of apoptotic cells [a]
					F-actin polymerization assay	-decreased percentage of F-actin polymerization [a]
					Protein expression	-upregulated: active caspase-3 [a]<br>-downregulated: Bcl-2 [a]
Badr et al. (2013) [38]	*Walterinnesia aegyptia*	[Xenograft] BALB/c mice (10 wk, 22-25g)<br>MDA-MB-231	2.5 μg/kg/d	28 d	Annexin V and PI assay	-increased percentage of early and late apoptotic cells [a]
					JC-1 dye assay	-decreased mitochondrial membrane potential and a subsequent increase in apoptosis [a]
	Walterinnesia aegyptia with silica NPs		1 μg/kg/d		CFSE assay	-reduced IGF-1-mediated proliferation of breast cancer cells [a]
					Oxidative stress marker	-increased ROS, hydroperoxide and nitric oxide
					Protein expression	-upregulated: caspase-3, -8, and -9 activities [a]<br>-upregulated: Bak, Bax, Bim, and cyclin B1<br>-downregulated: Bcl-2, Bcl-xL, Mcl-1, cyclin D1
Badr et al. (2014) [41]	*Walterinnesia aegyptia*	Human Breast cancer tissue samples	NR	12 h	Annexin V and PI assay	-increased percentage of apoptotic cells [a]
					Cell cycle analysis	-increased apoptosis induction and decreased the percentage of cells in S phase [a]
					CFSE assay	-decreased the percentage of proliferating cells [a]
					Oxidative stress marker	-increased ROS, hydroperoxide and nitric oxide

					Protein expression	-upregulated: caspase-3, -8, and -9 activities ^{a}
Jimenez-Canale et al. (2022) [29]	*Crotalus molossus molossus* with chitosan NPs	T-47D	7.81, 15.62, 31.25 μg/mL	30 min	Fluorescence Intensity assay	--reduced Fluorescence Intensity (at 7.81, 15.62 μg/mL$^{b)}$
Jokhio et al.(2005) [42]	*Cobra*	Human Breast cancerous tissues	10, 25, 50 μg/mL	NR	Spectrophotometric analysis	-inhibited the formation of nucleic acids (maximum effect at 25 μg/ml)
Malekara et al. (2020) [32]	*Vipera raddei kurdistanica*	MDA-MB-231 MCF-7	0.31, 0.62, 1.25, 2.5, 5, 10 μg/mL	48 h	TUNEL assay	-activated apoptosis in a concentration-dependent manner
					JC-10 dye assay	-decreased mitochondrial membrane potential (at 0.31 ^{a}, 0.62 ^{b}, 1.25 ^{b}, 2.5 ^{b}, 5 ^{b}, 10 ^{c} μg/mL in MCF-7cells, at 0.31 ^{a}, 0.62 ^{b}, 1.25 ^{b}, 2.5 ^{b}, 5 ^{b}, 10 ^{b} μg/mL in MDA-MB-231 cells)
					Cytochrome C Measurement	-increased cytochrome C (at 0.31 ^{b}, 0.62 ^{b}, 1.25 ^{b}, 2.5 ^{b}, 5 ^{c}, 10^{c} μg/mL in MCF-7 cells, at 0.31 ^{a}, 0.62 ^{b}, 1.25 ^{b}, 2.5 ^{b}, 5 ^{c}, 10 ^{c} μg/mL in MDA-MB-231 cells)
					Oxidative stress marker	-increased ROS
					Protein Expression	-upregulated: pro-apoptotic BAX protein -downregulated: anti-apoptotic BCL-2 protein
					Gene Expression	increased BAX mRNA and decreased BCL-2 mRNA (at 1.25 ^{b}, 2.5 ^{b}, 5 ^{b}, 10 ^{b} μg/mL)
Soliman et al. (2024) [39]	*Naja haje* with silica NPs	[Xenograft] Albino Wistar rats (7-9 wk, 100-120 g) MDA-MB-231	0.1, 0.02 mg/kg	28 d	Inflammatory cytokine	-decreased IL-6, TNF-*α* levels
					Gene Expression	-downregulated: anti-apoptotic gene BCL-2 levels -upregulated: P53, BAX, and Caspase-3 in G 2-5 ^{c} (maximum effect at 0.1 mg/kg)

^{a}−p<0.05; ^{b}−p<0.005; ^{c}−p<0.001; h−hours; min−minutes; d−days; wk−weeks; Bcl-2−B-cell lymphoma 2; Bim−Bcl-2 interacting mediator of cell death; CFSE−Carboxyfluorescein succinimidyl ester; IL-6−Interleukin 6; IL-8−Interleukin 8; Mcl-1−Myeloid cell leukemia-1; p-Erk1/2−Phosphorylated extracellular signal-regulated kinase 1/2; PI−propidium iodide; RhoC−Ras homolog gene family member C;

ROS–Reactive oxygen species; TNF-α–Tumor necrosis factor alpha; TUNEL–Terminal deoxynucleotidyl transferase dUTP nick end labeling; NR–Not reported

Table S4. Anticancer Mechanism of snake venom bioactive proteins in breast cancer cells.

Author (Year)	Snake Venom	Main component	Target cell	Concentration	Duration	Method	Results
Bezerra et al. (2019) [20]	*Bothrops jararacussu*	BthTX-I	MDA-MB-231, MCF-7, SK-BR-3	102 μg/mL	24 h	Annexin V and PI assay	-increased apoptosis of MCF-7 and SK-BR-3 cells [a] -increased necrosis of MCF-7 cells [a]
			MCF-7			Protein Expression	-upregulated: pro caspase-3, -8 and Beclin-1 [a] -downregulated: Bcl-2 [a]
Bhattacharya et al. (2023) [21]	*Russell's viper*	SPAD-1	MCF-7	0, 2, 20 nM	24, 48 h	Scratch assay	-restricted the invasive property of MCF-7 cells in a dose-dependent manner
				0, 95, 190, 385, 1920 nM	0.5, 1.5, 3 h	Cell dissociation assay	-increased cell detachment from the poly L-lysine-coated, laminin-coated and fibronectin-coated culture plate matrices
				0, 2, 20 nM	0, 24, 48 h	Invasion assay	-restricted the invasive property of MCF-7 cells in a dose-dependent manner
Bhowmik et al.(2017) [22]	*Naja kaouthia*	NKCT1 with gold NP	MCF-7	3.9 μg/ml	24 h	Annexin V and PI assay	-increased early and late apoptotic cells percentage
				3.9 μg/ml	24, 48, 72, 96 h	Boyden matrigel chamber assay Wound-healing assay	-decreased the level of cell invasion in a time-dependent manner -decreased cell motility in a time dependent manner
				3.9 μg/ml	24 h	Cell cycle analysis	-induced a G1 phase cell cycle arrest -decreased S phase (proliferative DNA content) -arrested in G0/G1 phase
				3.9 μg/ml	24, 48 h	Protein Expression	-inhibited cyclin D1-CDK4 on interruption of mitogenic signaling in MCF-7 cells -inactivated MAPK pathway by inhibiting the phosphorylation of ERK1/2 and p38 -downregulation of PI3K/Akt and NF-kB signaling
Derakhshani et al. (2020)	*Naja naja oxiana*	Recombinant Cytotoxin II	MCF-7	4 μg/mL	12, 24 h	Annexin V binding assay	-increased early and late apoptotic cells percentage

[23]					0, 12, 24 h	Wound-healing assay	-inhibited the migration of MCF-7 cells
					24 h	Gene Expression	-inhibited the migration of MCF-7 cells
Gallego-Londoño et al. (2025) [25]	*Crotalus durissus* *Naja atra*	Crotalicidin NA	MDA-MB-231 MCF-7	Crotalicidin: 12.5, 25, 50 μM/mL	6 h	Cell cycle analysis	-increased the number of cells in the sub-G1 phase (at 50 ^{c}, 12.5 ^{a} μM/mL in MCF-7 cells, at 25 ^{b}, 50 ^{c}, 6.25 ^{c}, 12.5 ^{c} μM/mL in MDA-MB-231 cells)
				NA: 3.125, 6.25, 12.5 μM /mL		fluorescence intensity assays	-did not lose mitochondrial membrane potential<br>-induced membrane leakage<br>-not involve the induction of apoptosis but is the loss of cell membrane integrity
				NA: 12.5 μM /mL		Cell membrane disruption analysis: confocal microscopy	-enhanced membrane permeability and cell death at higher concentrations
Gimenes et al. (2017) [26]	*Crotalus durissus collilineatus*	γCdcPLI	MDA-MB-231	25, 50 μg/mL	24 h	Annexin V and PI assay	-increased early and late apoptotic cells percentage (at 25 ^{c}, 50 ^{b} μg/mL)
				3.125, 6.25, 12.5, 25, 50 μg/mL		Adhesion assay	-inhibited adhesion in a dose-dependent manner
				25, 50 μg/mL		Mattrige transwell assay	-reduced invasion
				25 μg/mL		Wound healing assay	-inhibited migration
				25 μg/mL		Gene Expression	-upregulated: TNF<br>-downregulated: BAD, BAX, BCL2, BCL2L1, TNFRS10B ^{a}, TNFRSF1A ^{a} and CASP8 ^{c}<br>- Caspase-3 or -7 activation were not triggered
				25 μg/mL		Protein Expression	- increased the level of p-p53 level, p-ERK<br>-inhibited the activity of the PI3K/Akt pathway<br>-decreased the level of the p-Akt
			BALB/c mice (6 wk) Aortic	25, 50 μg/mL	7 d	Angiogenesis ex vivo assay	-reduced the number of sprouting elongated vessels

toxins

MDPI

			fragments				
			HUVEC	25, 50 μg/mL	18 h	Angiogenesis in vitro assay	-inhibited bFGF-induced vessel formation and decreased VEGF levels ^{c}
Hiu et al. (2021) [27]	*Naja sumatrana*	CTX-I	MCF-7	2, 4, 6, 8, 16, 32, 64 μg/mL	4, 8, 16, 24 h	Calcein-AM/ PI staining assay	-increased membrane permeabilization (with high CTX-I concentrations within a short period)
Jebali et al. (2014) [28]	*Macrovipera lebetina*	Lebecin	MDA-MB-231	25 μg/ml	2 h	Adhesion assays	-decreased attachment
				0, 5, 10, 15 μg/ml	5 h	Boyden chamber assays	-decreased migration in dose-dependent manner
				0, 5, 10, 15 μg/ml	5 d	Proliferation assays	- reduced the cells proliferation<br>- inhibition of proliferation reached 75% in 5 d
Latinovi et al. (2017) [31]	*Vipera ammodytes ammodytes*	Dimeric disintegrin	MDA-MB-231	2.5, 5.0, 7.5, 15.0 nM	1, 6, 12, 24 h	Wound healing assay	-decreased migration (at >2.5 nM ^{c})
Silva et al. (2018) [33]	*Bothrops pauloensis*	BnSP-6	MDA-MB-231<br>MCF-7	6.25, 12.5, 25, 50, 100 μg/mL	24 h	Genotoxicity: micronucleus assay	-increased themicronucleus incidence ratio in a dose-dependent manner (in MDA-MB-231 cells)
			MDA-MB-231	50 μg/mL	24 h	-TUNEL assay<br>-DNA fragmentation assay	-increased TUNEL-positive cells<br>-induced DNA damage
						Cell cycle analysis	-induced a reduction in 2N (G1) phase cells and accumulation in the G2/M phase
					24, 48, 72 h	Proliferation assay	-inhibited MDA-MB 231 proliferation both in the presence (24 ^{b}, 48 ^{d}, 72 ^{c} h) and absence of bFGF (24 ^{b}, 48 ^{d}, 72 ^{d} h)
					24 h	Gene Expression	-upregulated: CDH1<br>-downregulated: CCND1, CCNE1, CDC25A, CHEK 2, E2F1 and NF-kB
Swenson et al. (2004) [40]	*Agkistrodon contortrix contortrix*	contortrostatin	[Xenograft] Mice (5 wk)	30 μg, gd i.t.	8 wk	Angiogenesis assay: CD31 staining and	- intratumoral injection of contortrostatin resulted in an 82% reduction in neovascularization, while intravenous delivery
		liposomal		100 μg			

		contortrostatin	MDA-MB-231	twice/wk i.v.		hotspot quantification	of liposomal contortrostatin achieved a 94% reduction
Tsai et al. (2016) [34]	*Naja naja atra*	CTX III	MDA-MB-231	0-2 µg/mL	24 h	- Boyden chamber invasion<br>-Wound healing assay	- decreased migration and invasion
				2 µg/mL	24 h	EMT Marker Expression	-decreased expression of epithelial adherens junction proteins<br>-decrease of mesenchymal biomarkers, N-cadherin and Vimentin levels in EGF-treated cells<br>-enhanced expression of E-cadherin
				0, 0.5, 1, 2 µg/mL	24 h	Protein Expression	-inhibited MMP-9 expression<br>-suppressed the expression of nuclear and cytosolic NF-kB in EGF-induced cells<br>-inhibited the EGFR phosphorylation and downstream activation of PI3K/Akt and ERK1/2
Van Petten de Vasconcelos Azevedo et al. (2016) [35]	*Bothrops pauloensis*	BnSP-6	MDA-MB-231	10, 50 µg/mL	24 h	Annexin V-FITC and PI staining	-increased early and late apoptotic cells
				50 µg/mL	24 h	Fluorescence microscopy	-stimulated autophagic by the appearance of punctuate monodansylcadaverine fluorescence
				3.12, 6.25, 12.5, 25, 50 µg/mL	24 h	Cell adhesion assay	-inhibited the adhesion in a dose-dependent manner
				50 µg/mL	24 h	Wound-healing assay	-significantly decreased cellular migration
				50 µg/mL	24 h	Gene Expression	-upregulated: TNF, TNFRSF10B, TNFRSF1A and CASP8 genes<br>-downregulated: BCL2, BCL2L, BAX and BAD<br>-stimulated P53 and BRCA2 genes
Van Petten de Vasconcelos Azevedo et	*Bothrops jararacussu*	BthTX-II	MDA-MB-231	10, 50 µg/ml	24 h	Annexin V-FITC and PI staining	-increased late apoptosis > early apoptosis and increased necrosis (at 10 [c],50 [c] µg/ml)
				50 µg/ml	24 h	Fluorescence microscopy	-induced approximately 70% autophagy

toxins

MDPI

al. (2019) [36]				10, 50 μg/ml	24 h	Cell cycle analysis	-increased Sub-G0 cell cycle<br>- promoted G2-M cell-cycle arrest
				2.5, 10, 25, 50 μg/ml	30 min	Adhesion assays	-inhibited adhesion
				1, 10, 50 μg/ml	24 h	Matrigel-coated transwell assay	-inhibited invaion
				10, 50 μg/ml	24 h	-Wound healing assay<br>-Transwell migration assay	-reduced migration
				10, 50 μg/ml	24 h	EMT Marker Expression	-downregulated: MCAM, CTNNB1, and TWIST1<br>-upregulated: CDH1 (E-cadherin)<br>-increased E-cadherin<br>-decreased CK-5, Vimentin
				50 μg/ml	24 h	Gene expression	[cell death]<br>-upregulated: TNF and its receptor TNFRSF1A genes, CASP8/TP53<br>-downregulated: MDM2, ANGPT1<br>[Cell Proliferation]<br>-upregulated: ATM<br>-downregulated: CCND1, CCNE1, CDC25A, E2F1, AKT1 and AKT3 genes<br>-decreased integrins: α2, β1, αvβ<br>-increased tumor suppressor genes: BRCA1 and BRCA2
Van Petten de Vasconcelos Azevedo et al. (2022) [37]	*Bothrops jararacussu*	BthTX-II	HUVEC	10, 50 μg/mL	24 h	Cell Cycle Analysis	-decreased the cell population in G0/G1[d]<br>-promoted an arrest in G2-M phase[d]
				1.57, 3.15, 6.25, 12.5, 25, 50 μg/mL	40 min	Adhesion assays	-inhibited adhesion (at 1.57~12.5 [a], 25, 50 [c] μg/mL)
			HUVEC + MDA-MB-231	10, 50 μg/mL	24 h	-Co-culture Invasion assay	-inhibited invasion with or without growth factors EGF, bFGF [c]

				-3D co-culture assay	-exhibited an effect on the total inhibition of cell co-culture migration and proliferation (at 50 μg/mL)
	HUVEC	1, 10, 25, 50 μg/mL	24 h	Wound-healing Assay	-reduced migration [d]
		1, 10, 25, 50 μg/mL	24, 48, 72 h	Proliferation Assay	-decreased the proliferation [d]
		10, 50 μg/mL	30 min	Angiogenesis in vitro assay	-inhibited vessel formation even with the presence of bFGF [d]<br>-reduced VEGF levels [c]
	BALB/c mice (6 wk) Aortic fragments	10, 50 μg/mL	7 d	Angiogenesis ex vivo assay	-reduced the number of cellular microvessels<br>-reduced the percentage of angiogenesis (at 10 [c], 50 [d] μg/mL)
	Chick embryos (3 d)	10, 50 μg/mL	4 d	Angiogenesis in vivo assay	-reduced in vessel caliber, tumor size, and tumor weight

[a]–$p<0.05$; [b]–$p<0.005$; [c]–$p<0.001$; [d]–$p<0.0001$; h–hours; min–minutes; d–days; wk–weeks; ANGPT1–Angiopoietin 1; BCL2–B-cell lymphoma 2; bFGF–basic fibroblast growth factor; BRCA1–breast cancer type 1 susceptibility protein; CASP8–caspase 8; CCND1–cyclin D1; CCNE1–cyclin E1; CDH1–cadherin 1 (E-cadherin); CDK4–cyclin-dependent kinase 4; CDC25A–cell division cycle 25A; CHEK2–checkpoint kinase 2; CK-5–cytokeratin 5; CTNNB1–catenin beta 1 (β-catenin); EGF–epidermal growth factor; EGFR–epidermal growth factor receptor; EMT–epithelial to mesenchymal transition; ERK1/2–extracellular signal-regulated kinase 1/2; MCAM–melanoma cell adhesion molecule; MDM2–mouse double minute 2 homolog; NF-κB–nuclear factor kappa-light-chain-enhancer of activated B cells; PI3K/Akt–phosphatidylinositol 3-kinase/protein kinase B pathway; SPAD-1–Serine Proteinase Associated Disintegrin-1; TNF–tumor necrosis factor; TNFRSF10B–tumor necrosis factor receptor superfamily member 10B; TNFRSF1A–tumor necrosis factor receptor superfamily member 1A; TP53–tumor protein p53; TUNEL–terminal deoxynucleotidyl transferase dUTP nick end labeling; TWIST1–twist-related protein 1; VEGF–vascular endothelial growth factor
