# Supplementary material for: Anticancer Activity of Snake Venom Against Breast Cancer: A Scoping Review
Source: Toxins (Basel). 2025 Sep 25;17(10):477. doi: 10.3390/toxins17100477 (PMC12567882; doi:10.3390/toxins17100477)
Supplement: Supplementary file 1 [file toxins-17-00477-s001.zip › Supplementary Material S3. Table S6. Search terms used in each database.pdf]

toxins

MDPI

Anticancer Activity of Snake Venom Against Breast Cancer: A Scoping Review

Eun-Jin Kim [1], Jang-Kyung Park [2], Soo-Hyun Sung [3,*] and Hyun-Kyung Sung [4,*]

[1] Department of Pediatrics of Korean Medicine, Korean Medicine Hospital, Dongguk University Bundang Medical Center, Seongnam 13601, Republic of Korea; utopialimpid@naver.com
[2] Department of Korean Medicine Obstetrics and Gynecology, School of Korean Medicine, Pusan National University, Yangsan 50612, Republic of Korea; vivat314@pusan.ac.kr
[3] Department of Policy Development, National Institute of Korean Medicine Development, Seoul 04554, Republic of Korea
[4] Department of Education, College of Korean Medicine, Dongguk University, Gyeongju 38066, Republic of Korea
* Correspondence: koyote10010@nikom.or.kr (S.-H.S.); shksolar@dongguk.edu (H.-K.S.)

Table S6. Search terms used in each database and results.

Medline via PubMed (13.6.2025.)

.	Searches
#1	"breast neoplasms"[TIAB] OR "breast cancer"[TIAB] OR "breast tumor"[TIAB] OR "breast malignancy"[TIAB]
#2	"snake venom"[MH] OR "snake venom"[TIAB] OR "viper venom"[TIAB] OR "elapid venom"[TIAB] OR "cobra venom"[TIAB] OR "serpent venom"[TIAB] OR "snake toxin"[TIAB]
#3	"in vivo"[TIAB] OR "in vitro"[TIAB] OR "experimental study"[TIAB] OR "cell line"[MH] OR"cell line"[TIAB] OR "mouse model"[TIAB] OR "animal model"[TIAB]
**#4**	**#1 AND #2 AND #3**

Embase via Elsevier (13.6.2025.)

	Searches
#1	'breast tumor'/exp OR 'breast tumor':ab,ti OR 'breast cancer'/exp OR 'breast cancer':ab,ti OR 'breast neoplasm'/exp OR 'breast neoplasm':ab,ti OR 'breast malignancy'/exp OR 'breast malignancy':ab,ti
#2	'snake venom'/exp OR 'snake venom':ab,ti OR 'viper venom'/exp OR 'viper venom':ab,ti OR 'cobra venom'/exp OR 'cobra venom':ab,ti OR 'elapid venom'/exp OR 'elapid venom':ab,ti OR 'serpent venom'/exp OR 'serpent venom':ab,ti OR 'snake toxin'/exp OR 'snake toxin':ab,ti
#3	'in vivo'/exp OR 'in vivo':ab,ti OR 'in vitro'/exp OR 'in vitro':ab,ti OR 'experimental study'/exp OR 'experimental study':ab,ti OR 'cell line'/exp OR 'cell line':ab,ti OR 'animal model'/exp OR 'animal model':ab,ti
**#4**	**#1 AND #2 AND #3**

CENTRAL (13.6.2025.)

	Searches
#1	MeSH descriptor: [Breast Neoplasms] explode all trees
#2	((breast neoplasms) OR (breast cancer) OR (breast tumor) OR (breast malignancy)):ti,ab,kw
#3	#1 OR #2

#4	MeSH descriptor: [Snake Venoms] explode all trees
#5	((snake venom) OR (snake venom) OR (viper venom) OR (elapid venom) OR (cobra venom) OR (serpent venom) OR (snake toxin)):ti,ab,kw
#6	# 4 OR #5
#7	MeSH descriptor: [Models, Animal] explode all trees
#8	MeSH descriptor: [Cell Line] explode all trees
#9	((in vivo) OR (in vitro) OR (experimental study) OR (animal model) OR (cell line)):ti,ab,kw
#10	#7 OR #8 OR #9
**#11**	**#3 AND #6 AND #10**

CINAHL (EBSCOhost) (13.6.2025.)

	Searches
#1	MH "Breast Neoplasms"
#2	TX "breast cancer" OR "breast tumor" OR "breast malignancy"
#3	#1 OR #2
#4	MH “Snake Venoms”
#5	TX "viper venom" OR "elapid venom" OR "cobra venom" OR "serpent venom" "snake toxin"
#6	#4 OR #5
#7	MH "In Vivo Studies"
#8	MH "In Vitro Studies"
#9	MH "Experimental Studies"
#10	MH "Cell Line"
#11	MH "Animals"
#12	TX "in vivo" OR "in vitro" OR "experimental study" OR "cell line" OR "mouse model" OR "animal model"
#13	#7 OR #8 OR #9 OR #10 OR #11 OR #12
**#14**	**#3 AND #6 AND #13**

Korean medical databases - ScienceON, Korean traditional knowledge portal, Korea Citation Index, Research Information Sharing Service, OASIS, and Korean Medical database (13.6.2025.)

	Searches
#1	"breast neoplasms" OR "breast cancer" OR "breast tumor" OR "breast malignancy" OR " 유방암" OR "유방종양" OR "유방악성종양"
#2	"snake venom" OR "viper venom" OR "elapid venom" OR "cobra venom" OR "serpent venom" OR "snake toxin" OR "뱀독" OR "사독" OR "코브라독"
#3	"in vivo" OR "in vitro" OR "experimental study" OR "cell line" OR "mouse model" OR "animal model" OR "동물실험" OR "세포실험" OR "실험연구"
#4	#1 AND #2 AND #3
